# A generalized method for calculating plasmoelectric potential in non-Mie-resonant plasmonic systems

**DOI:** 10.1515/nanoph-2021-0610

**Published:** 2022-02-10

**Authors:** Yunkun Xu, Yulong Fan, Ye Ming Qing, Tie Jun Cui, Dangyuan Lei

**Affiliations:** Department of Materials Science and Engineering, City University of Hong Kong, 83 Tat Chee Avenue, Kowloon, Hong Kong, China; State Key Laboratory of Millimetre Waves, School of Information Science and Engineering, Southeast University, Nanjing 210096, China

**Keywords:** local heating, non-Mie-resonant system, plasmoelectric potential, plasmonic particle-on-film nanocavity

## Abstract

Since its first observation in 2014, plasmoelectric potential (PEP) has drawn a great deal of research interest in all-metal optoelectronics and photochemistry. As an optical thermodynamic phenomenon induced by the electron number dependent equilibrium temperature in plasmonic nanostructures, the early theoretical model developed for calculating PEP is only applicable to Mie-resonant nanostructures, such as a gold nanosphere on a conductive indium tin oxide (ITO) substrate, where the transfer efficiency of hot electrons from gold to ITO can be analytically determined. Without the presence of the substrate, the temperature increase on the gold nanosphere induced by plasmonic absorption was calculated on the basis of thermal radiation in vacuum, which probably over-estimates the actual temperature increase in comparison to realistic experimental conditions. Here, we propose an equilibrium-thermodynamics computational method to quantify the actual efficiency of plasmon-induced electron transfer between a non-Mie-resonant metallic nanostructure and a conductive substrate and hence determine the resultant plasmoelectric potential. With a less than 2.5% relative error in predicting the steady-state temperature of a Mie-resonant nanoparticle in vacuum, and a more strict evaluation of the plasmonic local heating induced temperature increase in a single plasmonic nanostructure or an array of such structures under continuous-wave illumination (CWI), our generalized method provides a robust and accurate approach for quantifying PEP in various plasmonic-particle (array)-on-film nanocavities.

## 1 Introduction

Different from hot carriers originating from the nonradiative plasmon decay in a metal nanostructure [1, 2], the PEP, resulting from charge transfer between the nanostructure and a conducting substrate, exhibits the maximum where the spectral dependence of light absorption is steepest yet becomes zero at the absorption peak [3, 4]. While plasmonic hot carriers have been broadly exploited in various plasmon-medicated photochemical reactions [1, 2, 5], the latter has only recently been recognized to play a prominent role as well [6], [7], [8], [9], [10], [11], [12], [13], [14], [15], [16], [17]. However, the early theoretical model developed by Atwater et al. [3, 4] for calculating the PEP is only applicable to Mie-resonant plasmonic nanostructures where the efficiency of the hot electron transfer between the nanostructure and the substrate can be analytically determined. This limitation has restricted the use of their model to spherical or ellipsoidal Mie-resonant nanostructures. In addition, heat radiation of the nanostructure in vacuum is used to determine the plasmonic heating induced temperature increase in the model [3, 4], which, to some extent, over-evaluates the actual temperature increase.

In this work, we make two important modifications to improve the application scope and the accuracy of the previous model. First, we introduce an “equivalent wavelength” method to determine the spectral dependence of absorption cross section (leading to the increase of local temperature) on the electron number of a metallic nanostructure by assuming that the absorption difference under optical illumination at two neighboring wavelengths mainly comes from their permittivity difference. If one can find another wavelength at which the permittivity can be considered the same as the wavelength of interest, the absorption cross section of the excited metallic nanostructure can be directly deduced by deriving its absorption spectrum without charge transfer. By comparing the results obtained by our modified method and the strict treatment with Mie theory, we demonstrate the introduced approximation only brings about a relative error of less than 2.5% in the local temperature increase. Second, we directly perform the straightforward heat transfer calculations with COMSOL Multiphysics to determine the plasmonic local heating induced temperature rise by a single metallic nanostructure in a more realistic way, and then apply the theoretical model to find the lattice-dependent mutual heating enhancement factor, which was firstly proposed by Baffou et al. [18], to account for the temperature increase contributed by all the nearby nanostructures. Therefore, our approach can be used to calculate more accurately the local temperature of an individual metallic nanostructure as well as a metallic nanostructure in an array. Based on these two modifications, we have successfully predicted the electron transfer induced PEP in more general plasmonic systems under CWI, including metallic particle-on-film nanocavities, plasmonic perfect absorbers, substrate-induced Fano resonant metallic nanostructures, and surface plasmon resonant arrays on conductive substrates.

## 2 A generalized method for calculating plasmoelectric potential

In the visible spectral range, the dielectric function of metals is dictated by the Lorentz–Drude model, which relates the bulk plasma frequency *ω*_p_ to the electron number density *n*_e_, as expressed in Eqs. (1) and (2):

(1) $$\begin{array}{l} \epsilon \left(\omega \right)=\epsilon_{\infty }-\frac{f_{0}\omega_{\text{p}}^{2}}{\omega^{2}+\text{i}\Gamma_{0}\omega }+\underset{j=1}{\sum }\frac{f_{j}\omega_{\text{p}}^{2}}{\omega^{2}+\text{i}\Gamma_{j}\omega -\omega_{j}^{2}} \end{array}$$

(2) $$\begin{array}{l} \omega_{\text{p}}=\sqrt{\frac{n_{\text{e}}e^{2}}{m^{*}\epsilon_{0}}} \end{array}$$

where *f*_0_ and Γ_0_ denote the amplitude and damping rate of the interband transitions, *f*_*j*_, *ω*_*j*_ and Γ_*j*_ are the amplitude, resonance frequency and damping rate of the *j*th Lorentz oscillator describing the intraband transition, *e* is the electron charge, m* is the effective electron mass, and *ε*_0_ is the vacuum permittivity.

Now let us consider a Mie-resonant plasmonic nanostructure electrically connected to the ground under optical excitation. Its plasmonic absorption will result in a significant temperature rise and thus modify the free energy of the system. As a result, charge transfer between the structure and the conducting substrate occurs in order to lower the system’s free energy. Along with the change in the electron number of the structure, Eq. (2) shows that its bulk plasma frequency will change accordingly, leading to the changes of the surface plasmon resonance frequency *ω*_plasmon_ (see Eq. (1)) and the absorption cross section according to Mie theory. This leads to the transition of the system to a new equilibrium temperature and a free energy in return. Therefore, the total number of electrons *N* in the plasmonic nanostructure varies with the system’s temperature *T*(*N*) and free energy *F*(*N*, *T*) under CWI. When the system reaches its thermodynamic equilibrium, the free energy *F*(*N*, *T*) is minimized, leading to the equilibrium condition:

(3) $$\begin{array}{l} \mu =S\left(T,N\right)\frac{\text{d}T\left(N\right)}{\text{d}N} \end{array}$$

where *μ* and *S* are the electrochemical potential and the entropy, respectively. To solve the electrochemical potential turns to seek the solution of *S* and *N*.

Since it has been shown that *μ* is dominated by the electrostatic potential, i.e., PEP, due to the charging of the metallic nanostructure [4], *μ* can be obtained from the self-capacitance *C*_e_ of the conductive nanostructure:

(4) $$\begin{array}{l} V_{\text{PEP}}\cdot e=\mu =\frac{\left(N-N_{0}\right)e^{2}}{C_{\text{e}}} \end{array}$$

where *N*_0_ is electron number of the uncharged metallic nanostructure, and *V*_PEP_ is the PEP. Moreover, the system’s entropy can be given by:

(5) $$\begin{array}{l} S\left(T,N\right)=\overset{N}{\underset{0}{\int }}\frac{\pi^{2}k_{\text{B}}^{2}T\left(N^{\prime },\lambda \right)}{6E_{\text{F}}\left(N^{\prime },V\right)}\text{d}N^{\prime }-3k_{\text{B}}A_{0}\text{ ln}\frac{\theta }{T\left(N\right)}+4k_{\text{B}}A_{0} \end{array}$$

where *k*_B_ is the Boltzmann’s constant, *E*_F_ is the Fermi energy, *θ* is the Debye temperature of the material, and *A*_0_ is the total number of atoms in the metallic nanostructure. The first term on the right-hand side is referred to as the electron entropy [19], which can be neglected in the present case.

### 2.1 A generalized method to determine the dependence of equilibrium temperature on the electron number

The remaining problem is to determine the dependence of equilibrium temperature on the electron number in the metallic nanostructure. Considering the ultrafast timescales of electron–electron scattering and electron–phonon scattering in metals [20], the difference between the electron temperature, phonon temperature and structure temperature is ignored in our case. Assuming that the input power *P*_in_ is balanced by the output power *P*_out_ in steady state, we can write:

(6) $$\begin{array}{l} P_{\text{in}}=I_{0}C_{\text{abs}}\left(N,\lambda_{0}\right)=P_{\text{out}}\left[T\left(N,\lambda_{0}\right)\right] \end{array}$$

where *I*_0_ is the incident light intensity and *C*_abs_ refers to the absorption cross section of the metallic nanostructure with a total number of electrons *N* under optical illumination at *λ*_0_. *P*_out_[*T*(*N*, *λ*_0_)] represents the heat transfer from the plasmonic nanostructure to its surroundings under thermodynamic equilibrium, and needs to be specifically treated in each case. Therefore, we can obtain the plasmoelectric potential of the metallic nanostructure by combining Eqs. (2)–(5) if the absorption cross section dependence on *N* and *λ*_0_ is known. However, since it generally lacks an analytical solution for the absorption cross section spectrum for non-Mie-resonant plasmonic nanostructures, one has to perform time-consuming full-wave electrodynamic simulations for each electron number *N* in order to obtain an accurate absorption spectrum to solve Eq. (5) [4, 21]. To accelerate the calculations, here, we propose an “equivalent wavelength” approximation method based on the assumption that the difference in the absorption cross sections of a metallic nanostructure under optical illumination at two adjacent wavelengths mainly comes from the difference in their permittivity *ε*.

According to Eq. (1), the permittivity depending on the electron number density and illumination frequency can be rewritten as:

(7) $$\begin{array}{l} \epsilon \left(\omega \right)=\frac{n}{n_{0}}\left(\epsilon \left(n_{0},\omega \right)-\epsilon_{\infty }\right)+\epsilon_{\infty } \end{array}$$

where *n*_0_ is the electron number density of the plasmonic nanostructure without charge transfer and $\epsilon_{\infty }$ is the permittivity at infinite frequency. It is evident that the change of *n* results in the variation of *ε* and hence modifies the absorption cross section. Therefore, it is reasonable to choose a wavelength *λ* close to *λ*_0_ while keeping *ε*(*n*, *λ*_0_) ≈ *ε*[*n*_0_, *λ*(*n*, *λ*_0_)] and assume *C*_abs_(*n*, *λ*_0_) ≈ *C*_abs_[*n*_0_, *λ*(*n*, *λ*_0_)]. Here, the equivalent wavelength *λ*(*n*, *λ*_0_) can be determined by minimizing the difference between *ε*(*n*, *λ*_0_) and *ε*[*n*_0_, *λ*(*n*, *λ*_0_)], which is defined as $\left|\epsilon \left(n,\lambda_{0}\right)-\epsilon \left[n_{0},\lambda \left(n,\lambda_{0}\right)\right]\right|$ . This means that the absorption spectrum of a charged metallic nanostructure can be directly deduced by deriving the absorption spectrum of the uncharged nanostructure by performing optical modelling with commercially available software. In this study, full-wave finite-element-method simulations with COMSOL Multiphysics 5.5, Radio Frequency module was used to simulate the absorption cross sections of our systems.

### 2.2 Comparison between the results obtained by Mie theory and our generalized method

Herein, we test the accuracy of our generalized method by comparing the PEP results of a Mie-resonant metallic nanostructure calculated with our method and the previous strict method using the Mie theory with Lorentz–Drude fitting parameters [4]. In this model test study, we consider that a silver nanosphere having a radius of 10 nm in vacuum is connected to the ground in some way under monochromatic illumination with power density *I*_0_ = 100 W/m^2^. Assuming that the only way the nanosphere can gain (loss) energy from (to) the vacuum is through thermal radiation, we can obtain the temperature dependence on the electron number from the below equation:

(8) $$\begin{array}{l} T\left(N,\lambda \right)={\left(\frac{C_{\text{abs}}\left(N,\lambda \right)I+\sigma A\eta T_{\text{amb}}^{4}}{\sigma A\eta }\right)}^{\frac{1}{4}} \end{array}$$

where *σ* is the Stefan–Boltzmann constant, *A* is the surface area of the nanosphere, and *η* is the emissivity (0.01 for bulk silver).

**Figure 1:**
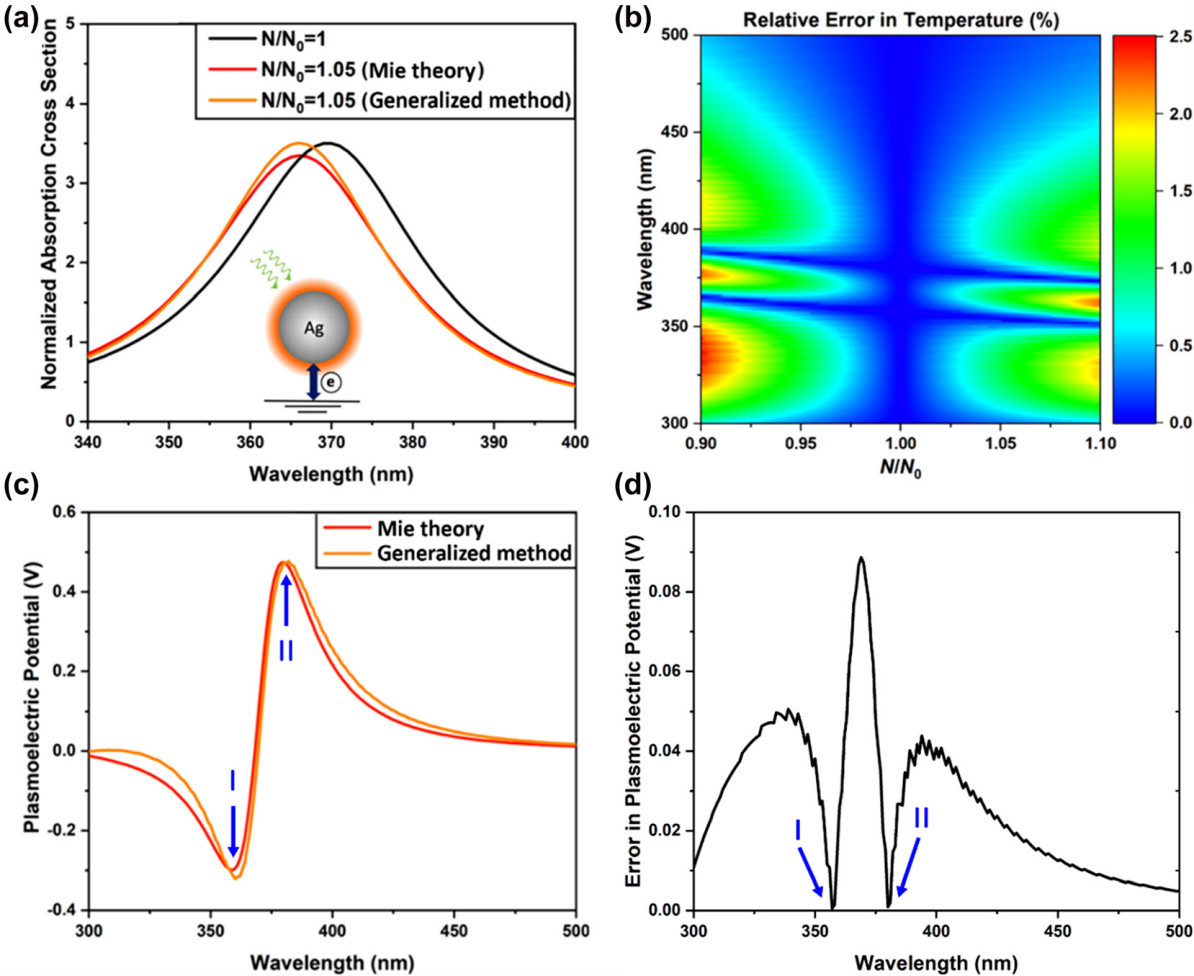
Validity test of generalized method in comparison with strict theoretical analysis. (a) Comparison between normalized absorption cross sections for a silver nanosphere of 10 nm in radius without and with charge transfer between the sphere and the ground (*N*/*N*_0_ > 1). (b) Relative error in temperatures derived from our generalized calculation method and the rigorous theoretical treatment. (c) Comparison between the PEP spectra calculated by the generalized calculation method and the rigorous theoretical treatment. (d) Error (absolute difference) in PEP obtained our generalized method compared to the rigorous theoretical treatment. “I” and “II” marked in (c) and (d) demonstrate the high accuracy of our method in predicting PEP at the wavelengths of interest for practical applications.

Figure 1a shows the calculated normalized absorption cross sections for the silver nanosphere. It is noted that our generalized approximation method gives reasonably accurate result (red curve) in the case of *N*/*N*_0_ = 1.05 as compared to the rigorous Mie theory result (orange curve), both giving the same resonance frequency. In this way, the electron number dependent equilibrium temperature can also be efficiently derived. Figure 1b displays the relative error, which is defined as the ratio of the absolute difference between the temperatures calculated by the two approaches and to the strict-theory predicted temperature, is less than 2.5% over a large spectral range and electron number variation, thereby verifying the validity of our generalized method within the optical range of our interest. Further, the PEP spectra obtained by both approaches in Figure 1c show nearly the same spectral dependence, except a small redshift of the result given by our generalized method (less than 5 nm). The absolute difference in PEP calculated by the two methods shown in Figure 1d is less than 0.05 V over the whole spectral range of our interest, where the largest deviation occurs in the PEP spectral range with the steepest slope near 395 nm. Moreover, it is noted that our method gives a nearly accurate value at the spectral valley (∼357 nm, marked as “I” in Figure 1c and d) and peak of PEP (∼380 nm, marked as “II” in Figure 1c and d), which can serve as working wavelengths of interest for practical applications. Therefore, the results shown in Figure 1 (as well as that in Figure S1 for the case of *N*/*N*_0_ = 0.95) verify the reliability and robustness of the proposed generalized calculation method for predicting PEP with high accuracy, thereby extending the applicability of the previous method to non-Mie-resonant nanostructures.

### 2.3 Accelerated calculations under the finite charge-transfer limit

Our generalized calculation method can be further accelerated by taking the finite charge-transfer limit. Considering the fact that the charge transferred from the nanostructure to the ground (substrate) or vice-versa is trivial compared to *N*_0_, we may assume that the entropy *S* and the differential of temperature d*T*/d*N* remain unchanged before and after charge transfer. Therefore, we can obtain the final expression of *μ*:

(9) $$\begin{array}{l} \mu =V_{\text{PEP}}\cdot e={S\frac{\text{d}T\left(N\right)}{\text{d}N}|}_{N=N_{0}} \end{array}$$

Since d*T*/d*N* and *T*(*λ*) can be easily obtained by our generalized calculation method, Newton’s method has been employed to solve the simultaneous Eqs. (3), (4) and (9) and the wavelength-dependent temperature rise which will be discussed in detail in the next section.

### 2.4 Accurate calculation of plasmonic heating induced temperature rise in metal film-coupled metallic nanoparticles and nanoparticle arrays

Plasmonic heating in single or arrayed metallic nanoparticles has been successfully investigated and experimentally verified by Baffou et al. in 2013 [18]. They used an effective dielectric thermal conductivity to evaluate the heating of a single plasmonic nanoparticle in steady state under CWI, which is referred to as the self-heating effect, and used a lattice geometry dependent parameter to evaluate the temperature increase contributed by surrounding nanoparticles to the one under study, which is the so-called mutual heating effect:

(10) $$\begin{array}{l} \Delta T=\Delta T_{\text{S}}\left(1+M\right) \end{array}$$

where Δ*T*_S_ signifies the temperature increase by self-heating, and *M* stands for the mutual heating parameter. Specifically, the self-heating component in the thermal steady state can be expressed as:

(11) $$\begin{array}{l} \Delta T_{\text{S}}=\frac{P_{\text{in}}}{4\pi \bar{k}\bar{R}} \end{array}$$

where *P*_in_ = *C*_abs_ *I*_0_ denotes the optical energy absorbed by the nanoparticle, $\bar{k}$ represents the effective thermal conductivity of the ambient, and $\bar{R}$ is the effective radius of the nanoparticle having the same volume [18, 22].

**Figure 2:**
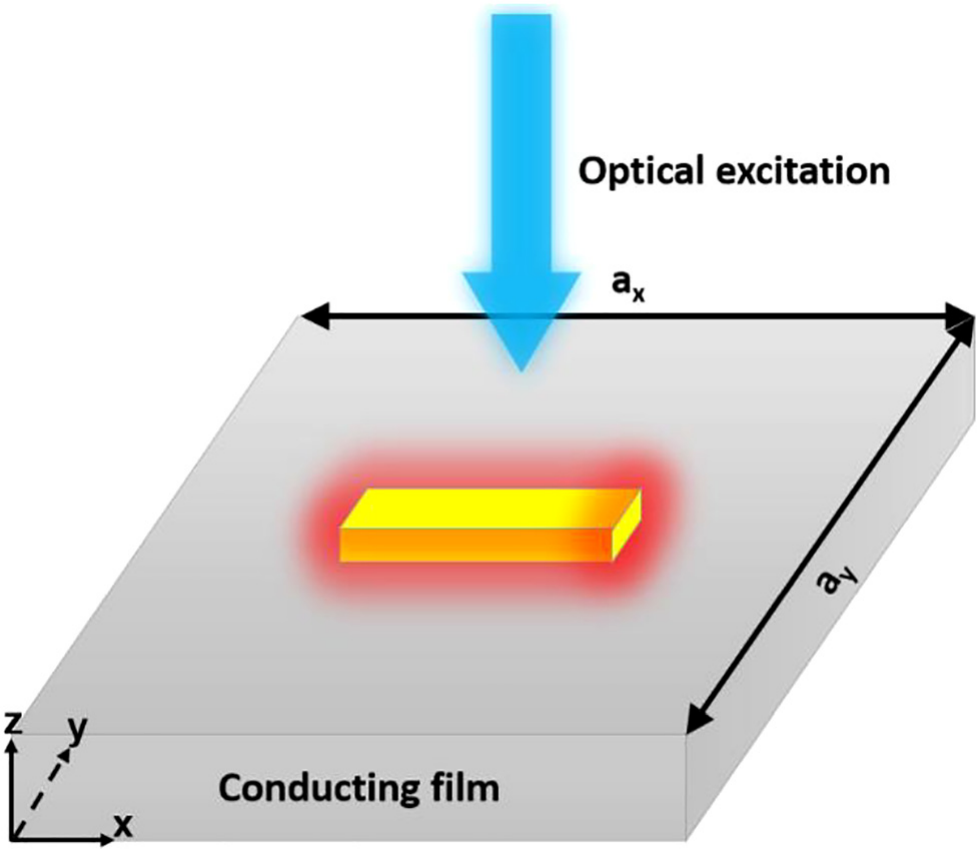
Schematic of plasmonic heating effect. An excited plasmonic nanoparticle is centered on a substrate with pitch periods being denoted as *a*_*x*_ and *a*_*y*_. Note that *a*_*x*_ and *a*_*y*_ are infinite for nanoparticle on film system and finite values for particle-array-on-film system. Here the nanoparticle is only represented by a yellow nanoscale cut wire (CW), while other nanostructures are not prohibited.

With regard to a plasmonic nanoparticle-array-on-film system, the lattice geometry dependent enhancement factor *M* should be determined for calculating the collective temperature increase. Take a square lattice (pitch period *a*_*x*_ and *a*_*y*_ in the *x*- and *y*-directions) under uniform circular-area illumination (diameter *D*) for example (see Figure 2). The mutual heating enhancement factor can be expressed as [18].

(12) $$M=\frac{\pi \bar{R}D}{a_{x}a_{y}}\left(1-\frac{2}{D}\sqrt{\frac{a_{x}a_{y}}{\pi }}\right)$$

When the diameter of the illumination spot is orders of magnitude larger than the square root of the unit-cell area, i.e., $D\gg \sqrt{a_{x}a_{y}}$ , *M* can be simplified as:

(13) $$M=\frac{\pi \bar{R}D}{a_{x}a_{y}}$$

Thus, the total temperature increase of the square lattice under the uniform circular-area illumination can be expressed as:

(14) $$\Delta T=\frac{q}{4\pi \bar{k}\bar{R}}\left(1+\frac{\pi \bar{R}D}{a_{x}a_{y}}\right)$$

However, we find the inaccuracy of effective thermal conductivity approximation proposed by Baffou et al. as well as the resultant self-heating temperature rise [18, 22], by carefully comparing the self-heating temperature rise with the numerical simulation results given by COMSOL Multiphysics 5.5, Heat Transfer in Solids module. More details about the simulation method and the thermal properties of all the related materials can be found in Section S2.1 in the Supporting Information. This discrepancy between the two results mainly results from the effective thermal conductivity approximation used in Eq. (11), and it significantly enlarges when the glass–water interface is replaced by the glass–air interface due to the giant thermal conductivity gap between glass (1.38 W/(m·K)) and air (0.033 W/(m·K)). Nevertheless, we find that the geometry dependent mutual heating enhancement factor, *M*, which accounts for the cumulative heats from all the other nanoparticles on the studied one, is still capable of correctly predicting the collective heating of arrayed nanoparticles because the inversely proportional relationship between the local temperature rise of the nanoparticle under study and its distance to the heating source still holds. In other words, Eqs. (11), (13), and (14) are valid once a proper effective thermal conductivity of the heating system is chosen (refer to Section S2.2 and 2.3 in Supporting Information for detailed discussion on the true effective thermal conductivity determination). As a result, in this study, we eventually decide to calculate the temperature rise profile of the nanostructure systems by performing the straightforward numerical simulation of the self-heating effect, and then using the mutual heating enhancement factor *M* to fast predict the collective heating effect. The relevant results of self-heating can be found in Figure S5 in Supporting Information with the relevant optical and thermal parameters given in Section 3.1–3.4.

## 3 Versatility and robustness tests in general models

To further test the versatility and robustness of our generalized calculation method of PEP, thereby shedding light on its benefits in potential applications of photo-electro-catalysis under diverse scenarios, we present in the following text the PEP arising from giant absorption of particle on conducting film systems calculated by using our proposed generalized method.

### 3.1 PEP induced by hybridized plasmonic mode from gold dimers

**Figure 3:**
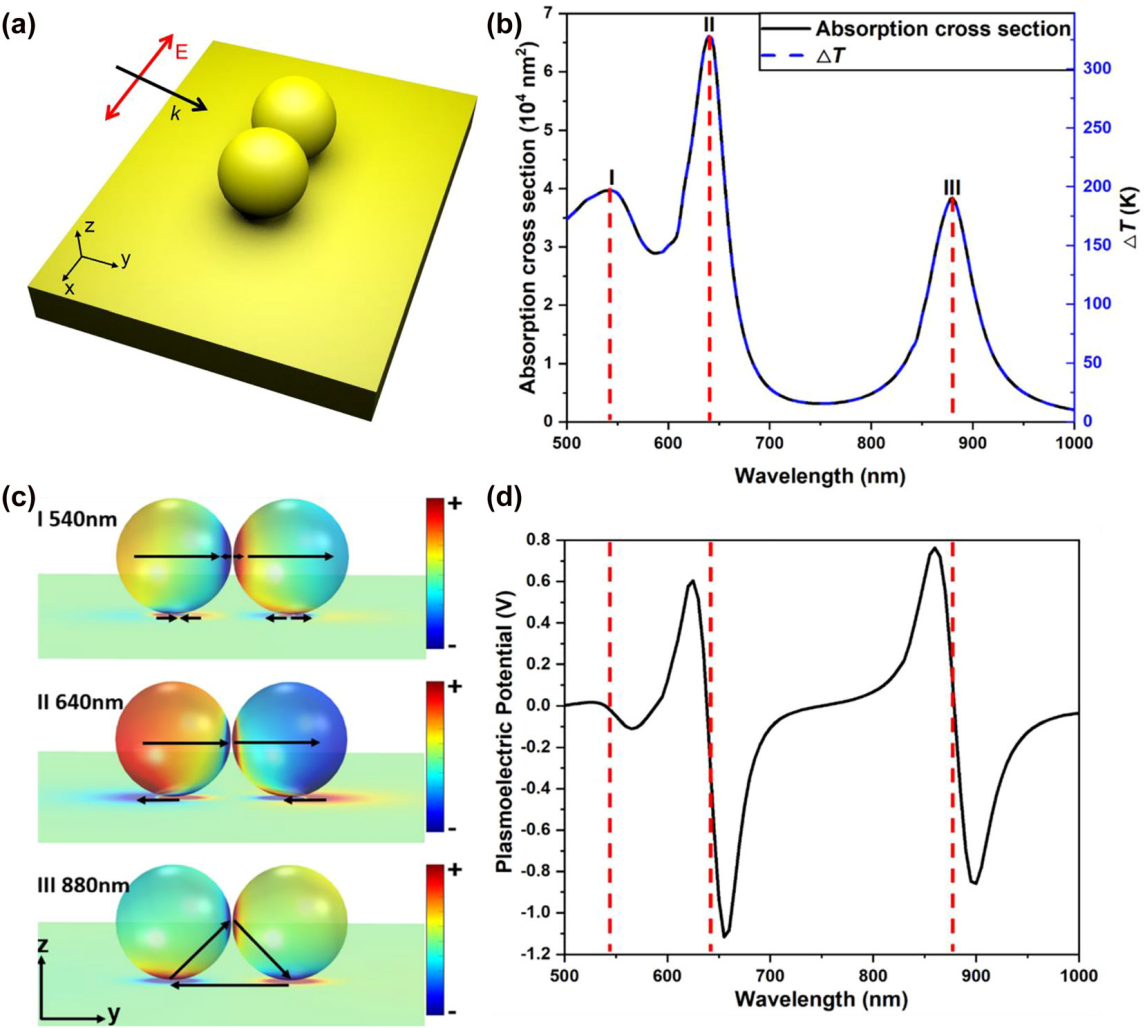
Temperature rise and PEP induced by hybridized plasmonic mode from gold dimers. (a) Schematic, (b) absorption spectrum (left axis) and temperature rise (right axis) of a gold nanosphere dimer coupled to a gold film. Each gold nanosphere is 50 nm in radius and coated by a 1 nm thick citric acid, resulting in an interparticle gap of 1 nm and a particle-film distance of 1 nm. (c) Simulated surface charge distribution at the three plasmon modes I, II, and III marked in (b). (d) Calculated PEP spectrum of the system in (a) under optical illumination with incident optical intensity 0.1 MW/cm^2^.

The gap mode in nanosphere dimer on metallic film is promising to enhance the plasmonic absorption induced temperature increase by a pronounced large absorption cross section in both visible and infrared region [23]. We model a gold dimer on gold substrate whose radius is 50 nm with a 1 nm interparticle gap. Meanwhile, a 1 nm gap between the dimer and the substrate was set to simplify the calculation. The optical property of gold is obtained by interpolating Johnson and Christy’s experimental data. The dimer is excited by incident waves from 53° with respect to the normal of the gold film. The optical polarization is along the long axis of the dimer, i.e., the *x*-axis. We schematically show this dimer on film system in Figure 3a, the corresponding absorption cross section (left coordinate) and temperature increase (right coordinate) in Figure 3b and the three typical modes corresponding to the three peaks marked as I, II and III in Figure 3c. Absorption peaks occur near *λ* = 540 nm, 640 nm, and 880 nm when the film-bonding gap-modes are excited.

To calculate the PEP here, we assume that the unique channel in which the nanospheres can exchange heat with the environment is through thermal conduction. The incident optical illumination intensity is set at 0.1 MW/cm^2^. Meanwhile, to simplify the problem, we assume that the electrostatic potential contains only the self-potential component. Calculated PEP results are shown in Figure 3d. Distinct from typical Mie scattering of plasmonic nanostructures, film coupled gap-modes endow the system with multiple PEP peaks ranging from 500 to 1000 nm. The wavelengths where the PEP achieves its peak and valley values at the steepest slope on the absorption spectrum just before and after the absorption peaks, agree well with the typical characteristics predicted by the previous theoretical analyses [4, 21].

### 3.2 Strong symmetric PEP induced by guided mode from perfect absorbers

A perfect plasmonic absorber on a metallic film efficiently converts incident optical energy into thermal dissipation that leads to a large self-heating effect owing to the large normalized absorption cross section, which can reach up to 30 [24]. The array-arranged perfect absorbers [25] may further largely increase the PEP due to large temperature increase due to the mutual-heating effect [18]. On the other hand, this array arrangement will also largely facilitate the control of the morphology, localization and rotation of the metallic cubes by conventional top–down fabrication strategy and may benefit the *in situ* monitoring of PEP photocatalysis. To this end, we model here a periodic silver cubes on silver film to reveal the giant absorption induced PEP effect. The optical property of silver is obtained by interpolating Johnson and Christy’s experimental data.

**Figure 4:**
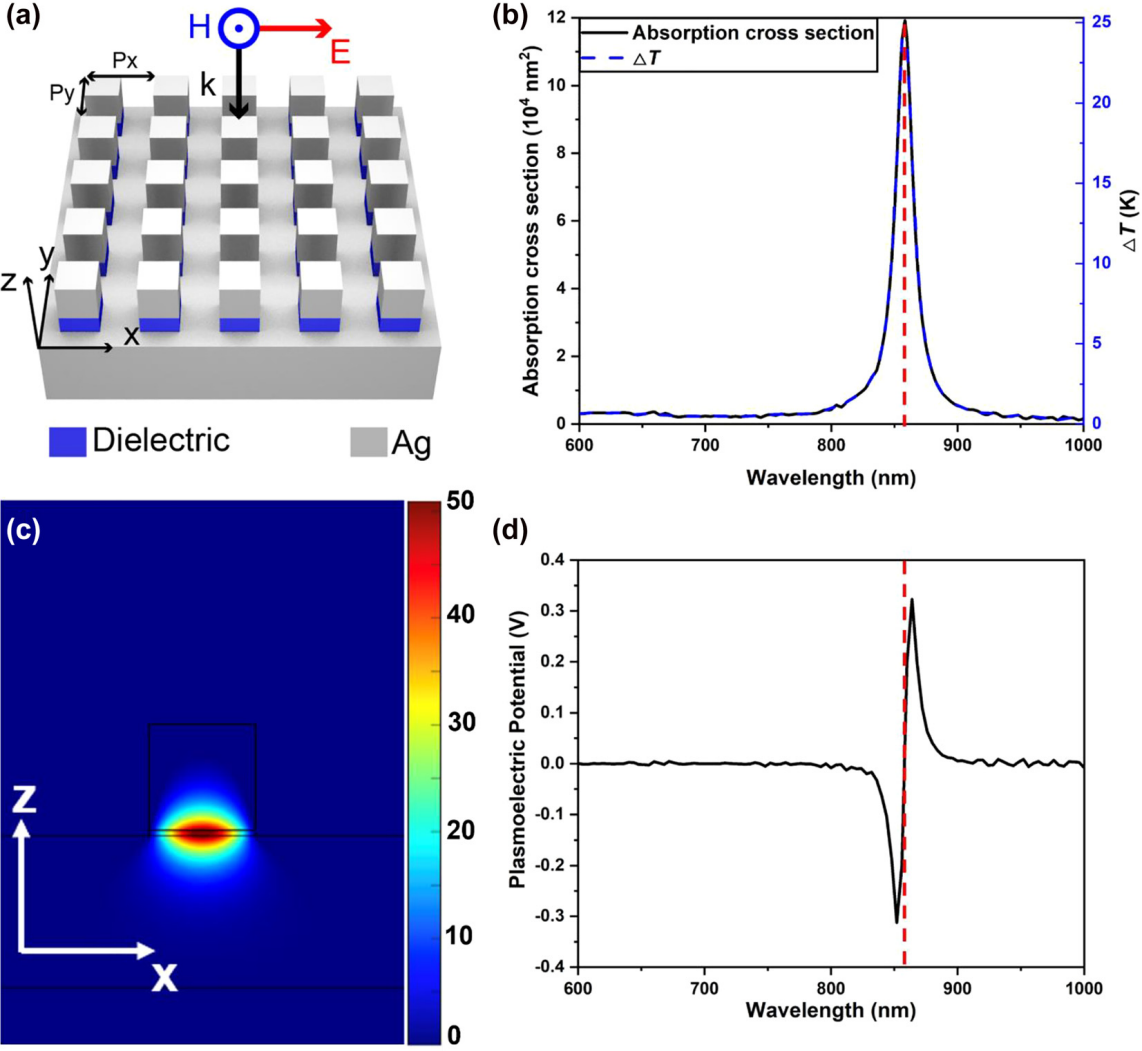
Temperature rise and PEP induced by guided mode from perfect absorbers. (a) Schematic of an array of 70 nm × 70 nm × 70 nm silver nanocubes separated from a silver substrate by a 4 nm thick dielectric spacer (in blue). The pitch period is 350 nm in both *x*- and *y*-directions. (b) Simulated absorption cross-section spectrum (left axis) and corresponding temperature rise (right axis) for the system in (a). (c) Simulated magnetic field distribution for the gap-plasmon guided mode at *λ* = 860 nm which is marked by dashed red lines in (b) and (d). The color map represents |*H*/*H*_0_| where *H*_0_ is the magnitude of the incident magnetic field component. (d) Calculated PEP spectrum for the system in (a) under an illumination spot of 0.2 cm in diameter and uniform intensity of 200 W/cm^2^.

In Figure 4a, we place arrayed 70 nm × 70 nm × 70 nm silver cubes on a silver substrate with 350 nm × 350 nm pitch period and spaced by an intermediate dielectric layer with *n* = 1.54 and thickness *g* = 4 nm. Incident waves normally impinge on the top of the silver cube with its electric field parallel to the side of the silver cube. Its absorption cross section spectrum (left coordinate) and the corresponding temperature increase (right coordinate) are shown in Figure 4b, where a near-unity giant absorbance (0.93 as shown in Figure S2) can be found when the gap-plasmon guided mode is excited (see in Figure 4c). Divided by the superficial area of the silver cube, we find that the normalized absorption cross section of one individual structure is as high as 23.2, implying that all incident energy can be totally absorbed and converted into heat by the cubes if more than 4.3% area of the silver film is occupied by such silver cubes.

The PEP calculated using our generalized method are shown in Figure 4d, with optical intensity 200 W/cm^2^ and diameter of optical illumination 0.2 cm. As shown in Figure 4d, a neat plasmoelectric valley and peak occur at the vicinity of the absorption peak in the absorption spectrum, while the PEP can be considered zero at other wavelength range if minor fluctuations are ignored. The results gained from our generalized calculation method agree well with the trends predicted by the theoretical analysis.

### 3.3 Strong asymmetric PEP stemming from substrate induced Fano resonance

The substrate-induced Fano resonance, as the result of interaction between dark quadrupolar and bright dipolar mode mediated by the introduced dielectric substrate, orientating predominantly on the vacuum side, can endow a silver cube with strong absorption at the Fano resonance when the silver cube is loaded on a dielectric substrate [26, 27]. Unlike the perfect absorber presented above, where an intermediate dielectric layer is necessarily required to support the guided mode, Fano resonance can sustain even when the gap layer is removed.

**Figure 5:**
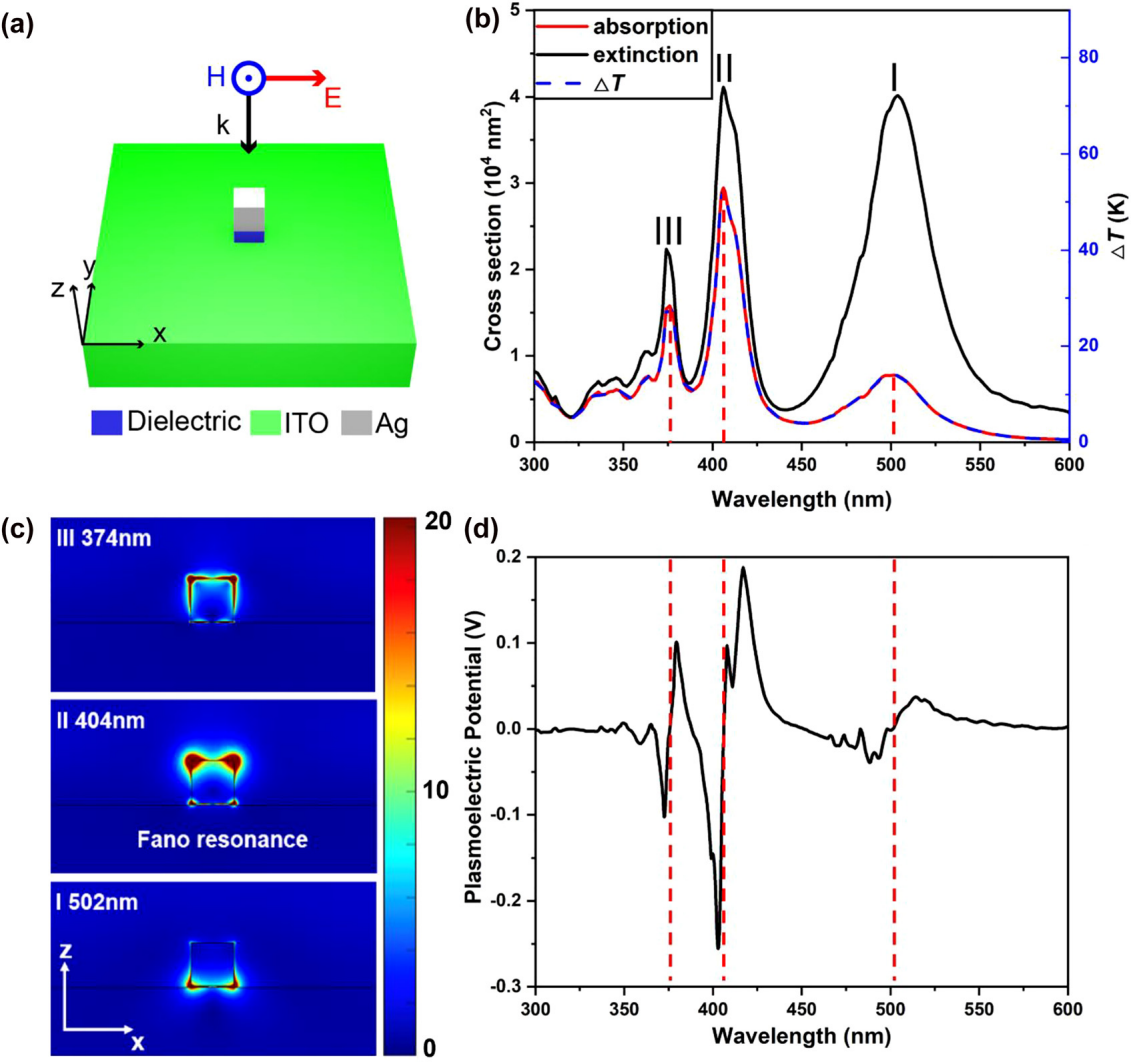
Temperature rise and PEP induced by substrate induced Fano resonance. (a) Schematic of a 60 nm × 60 nm × 60 nm silver nanocube loaded on an ITO substrate separated by a 1 nm thick citric acid spacer (blue), under *x*-polarized normal incidence. (b) Simulated absorption (red) and extinction (black) cross-section spectra (left axis) and corresponding temperature rise (right axis) for the system in (a). (c) Simulated electric near-field intensity at the three plasmon modes at 374 nm, 404 nm, and 502 nm, corresponding respectively to peaks III, II, and I in (b). (d) Calculated PEP spectrum for the system in (a) under optical illumination at intensity 0.1 MW/cm^2^.

We first model the Fano resonance to quantify the intense PEP introduced by Fano resonance when excited. In this simulation, a single silver nanocube with 60 nm side length is loaded onto an ITO substrate separated by a 1 nm thick Al_2_O_3_ spacer. Figure 5a shows the schematic of our simulation, where the incident excitation normally impinges on the silver cube with its polarization along the *x*-axis. *g* = 1 nm is chosen in our simulation to simplify the meshing of COMSOL as our silver cube is slightly smoothened with a spherical surface. The optical property of ITO is obtained by interpolating experimental data measured by König et al. [28]. The corresponding absorption (red) and extinction (black) cross sections (left coordinate) of this system along with its temperature increase (right coordinate) is presented in Figure 5b, showing three predominant resonance mode (marked as “I”, “II”, and “III” in the figure) in the wavelength range of interest and a temperature increase about 50 K due to the low thermal conductivity of ITO, in comparison to results shown in Figure 4b despite the larger absorption cross section of the perfect absorber. We show in Figure 5c the normalized |*E*/*E*_0_| profile at the surface of the silver cube, where *E*_0_ stands for the magnitude of incident electric field. We find typical Fano resonance at *λ* = 404 nm, where the quadrupolar mode orientates dominantly on the vacuum side, and a dipolar mode at *λ* = 502 nm, where the dipolar mode orientates dominantly on the substrate side. Additionally, the mode at *λ* = 374 nm refers to one of the primitive modes of the silver cube [26].

Meanwhile, a smoothing spline fitting with *R* ^2^ = 0.9975 is used to avoid the unexpected influence on the calculation results introduced by the tiny fluctuations in the simulated data. Calculated PEP results are shown in Figure 5d. Comparing the temperature increase and the resultant PEP of substrate induced Fano effect with the perfect absorber, we can infer that, apart from the slope of the absorption spectrum, the increased temperature also significantly contributes to the resultant PEP. This implies that the ITO substrate other than metallic substrate may be an excellent candidate in terms of thermal materials for achieving giant temperature rises.

### 3.4 Giant Dirac-like PEP resulting from plasmonic lattice mode

Now we move to the plasmonic lattice mode, which exhibits a Dirac-like absorption spectrum due to its Dirac-like absorbance at several discrete wavelength (see Figure S3) [29, 30]. The ultra-steep slope of lattice mode on ITO is promising to generate PEP orders of magnitude larger than all the configurations discussed above. Here we choose a 2D rectangular lattice array with periods of 600 nm in the *x*-axis and 300 nm in the *y*-axis, containing a 415 nm × 85 nm × 38 nm gold CW in each unit cell loaded on an ITO substrate and coated by a silica superstrate. The refractive index of silica is set to 1.45.

**Figure 6:**
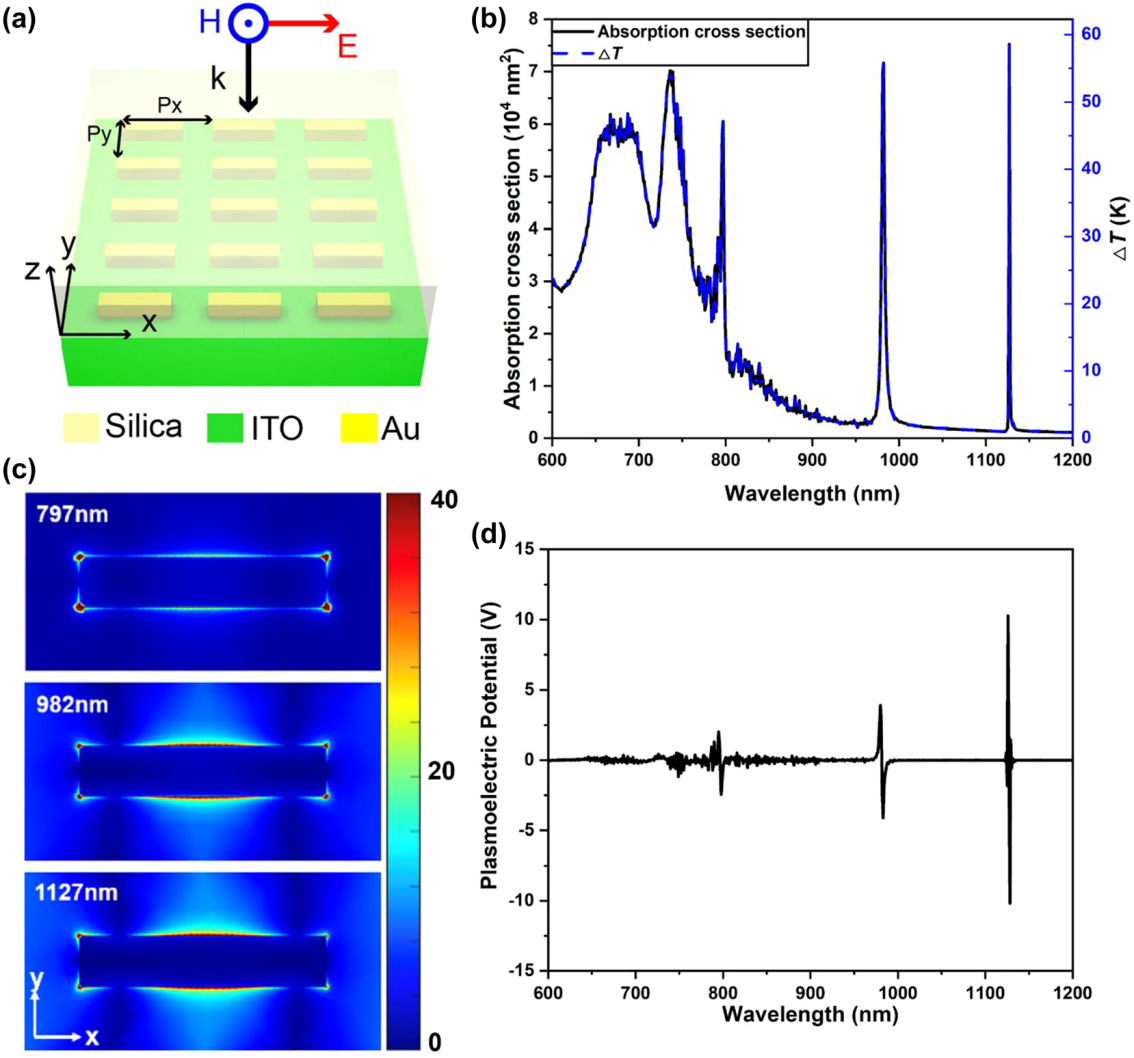
Temperature rise and PEP induced by plasmonic lattice mode. (a) Schematic of an array of 415 nm × 85 nm × 38 nm gold cut wires loaded on an ITO substrate and covered by a silica superstrate. The array has a period of 600 nm in the *x*-direction and 300 nm in the *y*-direction. (b) Simulated absorption cross-section spectrum (left axis) and calculated corresponding temperature rise (right axis) for the system in (a). (c) Simulated electric near-field intensity distributions at the three lattice modes located at 797 nm, 982 nm, and 1127 nm in the long wavelength range of (b). (d) Calculated PEP spectrum of the system with a rapid calculation method under the finite charge transfer approximation under an illumination spot of 0.2 cm diameter and uniform intensity of 200 W/cm^2^.

The structure of the unit cell of plasmonic lattice mode is schematically shown in Figure 6a, with normal incidence of optical excitation and optical polarization along the long axis of gold CW. The absorption cross section of this rectangular arrayed gold CWs with three sharp absorption peaks, i.e., three lattice modes, at 797 nm, 982 nm, and 1127 nm can be easily found in Figure 6b. The corresponding in-plane lattice mode resonance is shown in Figure 6c, displaying three lattice mode resonances with different strengths. Since the self-capacitance of this structure is hard to be theoretically determined in this model, we use the rapid solution here to give an approximate value of the PEP. The incident illumination intensity is chosen as 200 W/cm^2^, and the diameter of uniform circular illumination is chosen as 0.2 cm. The Dirac-like sharp and giant PEPs are shown in Figure 6d, resulting from the extremely sharp absorption peak and the more than 70 K temperature increase achieved at three absorption peaks. The giant PEP generated by lattice mode on ITO is also suited for working under bio-safety optical illumination, for example, *I*_0_ = 1 W/cm^2^. Whereas it needs further experimental support to verify our theoretical predictions.

## 4 Conclusions

In this work, we have developed an equilibrium-thermodynamics computational method to quantify the efficiency of plasmon-induced electron transfer between a metallic nanostructure and a conductive substrate and hence the resultant PEP. The results obtained by our novel yet rapid and generalized method compared with that gained from the strict theoretical analysis demonstrate the high accuracy of our method to approximate the absorption cross section and the temperature increase of a charged Mie-resonant plasmonic nanostructure illuminated in vacuum, and consequently obtain the resultant PEP. Then, the plasmonic local heating induced temperature increase under ambient conditions was incorporated into our proposed method. We successfully extend the application scope to various morphologies on film systems by testing our method to calculate the PEP generated by, for example, plasmonic particle-on-film nanocavities, perfect plasmonic absorber, Fano resonance metallic nanostructures and surface plasmon resonance array. We find that the peaks and valleys of PEP occur at the steepest slope on the absorption curve at the vicinity of absorption peaks, which agree well with the findings in previously published literature. We also find that a conductive substrate with lower thermal conductivity may hopefully lead to a much higher temperature increase of nanoparticles, implying that ITO is a promising candidate for PEP photochemical experiment. Finally, we believe that our method can even be applied to quantify the PEP of plasmonic nanoparticles without definitive self-capacitance working under extreme conditions, e.g., surface plasmon resonance array. However, the Dirac-like PEP resulted from the giant and sharp absorption spectrum need to be further experimentally supported. Overall, our equilibrium thermodynamics based computational method allows fast, robust and accurate calculation of PEP created between a metal nanostructure and a conductive substrate upon plasmon excitation, and this generalized method can be applied to various non-Mie-resonant nanoparticle (array) on film systems by once performing full-wave numerical simulations. Our method can thus be applied to predict the level of PEP and guide the photochemical catalysis experiment for future studies.

## Supplementary Material

Supplementary Material
