# Supplementary material for: A generalized method for calculating plasmoelectric potential in non-Mie-resonant plasmonic systems
Source: Nanophotonics. 2022 Feb 10;11(11):2453–64. doi: 10.1515/nanoph-2021-0610 (PMC11501218; doi:10.1515/nanoph-2021-0610)
Supplement: Supplementary file 1 — Supplementary Material [file j_nanoph-2021-0610_suppl.docx]

# Supporting Information for “A Generalized Method for Determining Plasmoelectric Potential in Non-Mie-Resonant Plasmonic Systems”

Yunkun Xu,^1‡^ Yulong Fan, ^1‡^ Ye Ming Qing,^2^ Tie Jun Cui,^2^ Dangyuan Lei^1^*

^1^Department of Materials Science and Engineering, City University of Hong Kong, 83 Tat Chee Avenue, Kowloon, Hong Kong, China

^2^State Key Laboratory of Millimeter Waves, School of Information Science and Engineering, Southeast University, Nanjing 210096, China

## S1 Supporting Figures for Main Text


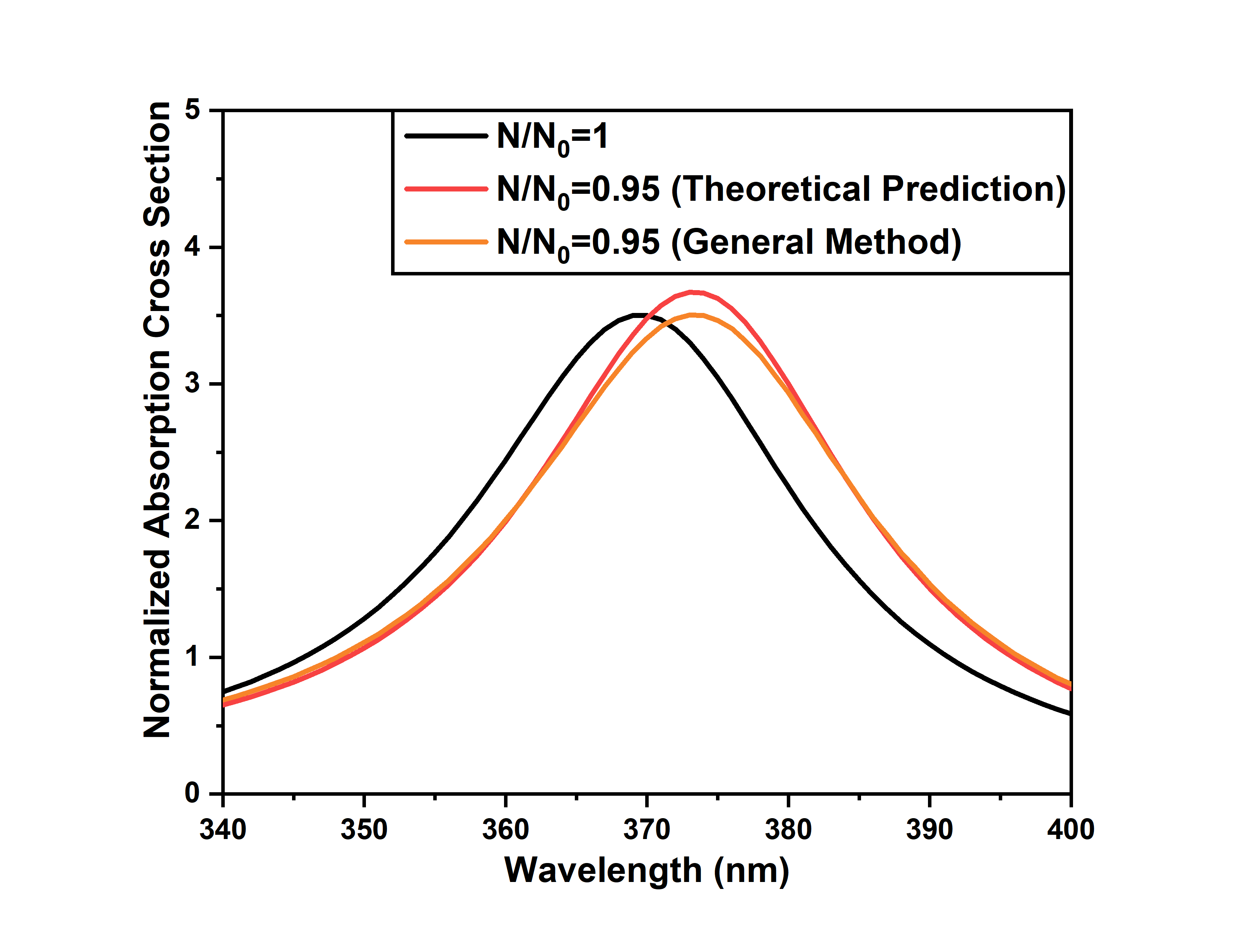


Figure S1. Comparison between the normalized absorption cross section of a silver nanosphere of radius 10 nm with and without charge transfer (*N*/*N*_0_<1).


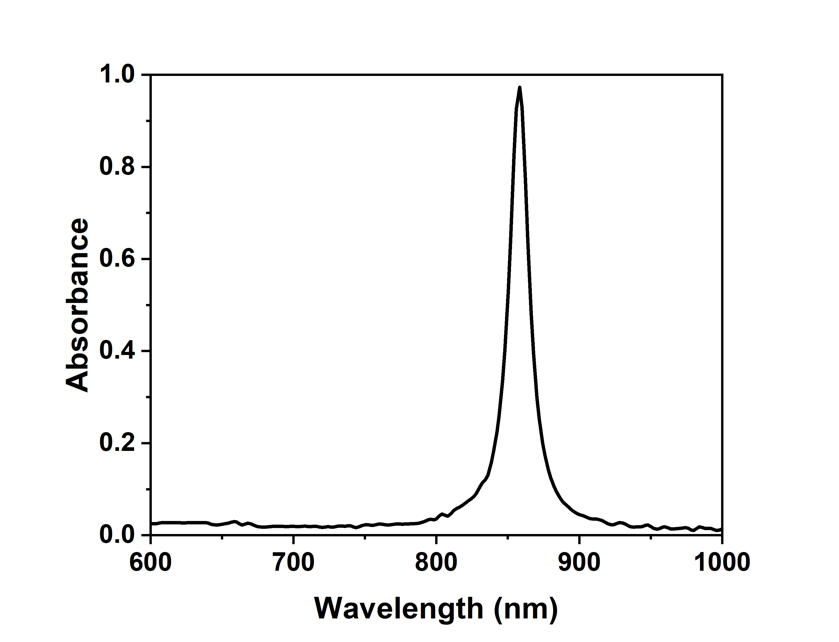


Figure S2. The absorbance spectrum of the perfect absorber used to deduce the absorption cross section of an individual perfect absorber as shown in Fig. 4b.


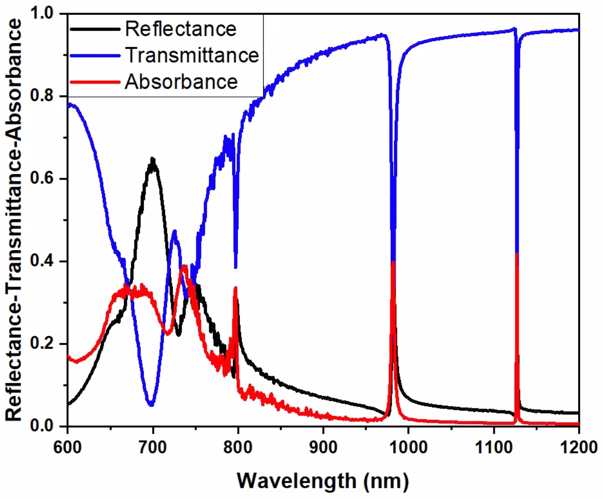


Figure S3. The reflectance, transmittance, and absorbance spectra of arrayed gold cutwires in silica on ITO substrate, whose structure is shown in Fig. 6(a).

**S2 Limitation of Baffou *et al*.’s Approximation and Accurate Determination of the Self-Heating Effect**

The self-heating-induced temperature increase is crucial for predicting the resultant collective heating effect in arrayed nanoparticles. By performing thermal simulations with the Heat Transfer module of COMSOL Multiphysics 5.5, we have found a limitation of Baffou *et al*.’s approximation in predicting the self-heating effect in plasmonic nanostructures, especially in the presence of a conducting substrate-air interface. Fortunately, the lattice geometry-dependent mutual heating enhancement factor, *M*, still holds true. Therefore, we can use the numerically calculated self-heating results to predict the ultimate collective heating effect and also retrieve the correct effective thermal conductivity in comparison with the results given by Baffou *et al*.’s method which obviously underestimates the impact of lattice geometry on the effective thermal conductivity.

S2.1 COMSOL modeling

Our thermal simulations are performed with the Heat Transfer in Solids module built in COMSOL Multiphysics 5.5. Assuming no forced convection in our system, we can ignore the convective heat transfer in air due to the minor stationary temperature difference induced by this process.[S1] Specifically, we model a single plasmonic nanostructure located at the center of a sphere with a radius of 2.5 mm, which is orders of magnitude larger than the nanostructure itself. The top half of the sphere is filled with air, and the bottom half is the substrate. The constant-temperature condition, i.e., at 293.15 K, is applied at the surface of the sphere to mimic the infinite ambient environment. The plasmonic nanostructure is chosen as heating source, and its dissipating power is determined by the product of its absorption cross section (obtained from optical modeling) and the incident optical intensity. Thermal properties of the relevant materials are summarized in Tab. S1. It should be noted that the gap between the single plasmonic nanostructure and the substrate is filled with a solid citric acid layer, whose thermal properties are also summarized in the Tab. S1.

Table S1. Heat transfer properties of all related materials used in COMSOL simulations.

| Material | Thermal Conductivity (W/(m·K)) | Density (kg/m^3^) | Heat capacity (J/(kg·K)) |
| --- | --- | --- | --- |
| Air | 0.033 | 0.898 | 1014 |
| Water | 0.6 | 1000 | 4200 |
| ITO | 10.2 | 7140 | 315 |
| Silver | 429 | 10940 | 237 |
| Silica | 1.38 | 2500 | 840 |
| Gold | 317 | 19300 | 129 |
| Aluminium oxide | Al_2_O_3_ (alpha) [solid, sapphire] (provided by COMSOL, temperature-dependent) | | |
| Citric acid | 0.25 | 1665 | 226.51 |

S2.2 Investigation of the validity of the theoretical model proposed by Baffou *et al*.[S2]

We have taken the same parameters used in Ref. [S2] to investigate the validity of the original model proposed by Baffou *et al*. As shown in Figs. S4a and c, we have obtained almost the same results when the five gold nanospheres are half-buried in glass and immersed in water, which means that Baffou *et al*.’s model works well for this structure configuration. However, when the particles are located on the glass surface and immersed in water, their heating properties revealed in Figs. S4b and d are almost the same as that shown in Figs. S4a and c, except a higher temperature rise within the gold nanoparticles. This observation originates from the fact that the thermal conductivity of water is lower (0.6 J/(m·K)) than that of glass (1.38 J/(m·K)), and it implies that the presence of the glass-water interface changes the temperature on the nanoparticles. By comparing the results shown in Figs. S4c and S4d, we can conclude that the self-heating induced temperature rise given by Eq. 11 and the geometry-dependent mutual-heating induced enhancement given by Eq. 13 still hold, provided that an appropriate effective conductivity (different from the proposal by Baffou *et al*.) is chosen. As a result, Eq. 14 is still robust and efficient for evaluating the collective-heating induced temperature rise of an array of plasmonic nanoparticles. It should be noted that a 1 nm thick citric acid layer with thermal properties given in Tab. S1 has been coated on the surface of each gold nanoparticle. However, we find this coating barely changes the actual temperature rise compared to that of bare nanospheres in water (results not shown here), due to the very thin layer of citric acid and its thermal conductivity (0.25 J/(m·K) comparable to that of water (0.6 J/(m·K)).


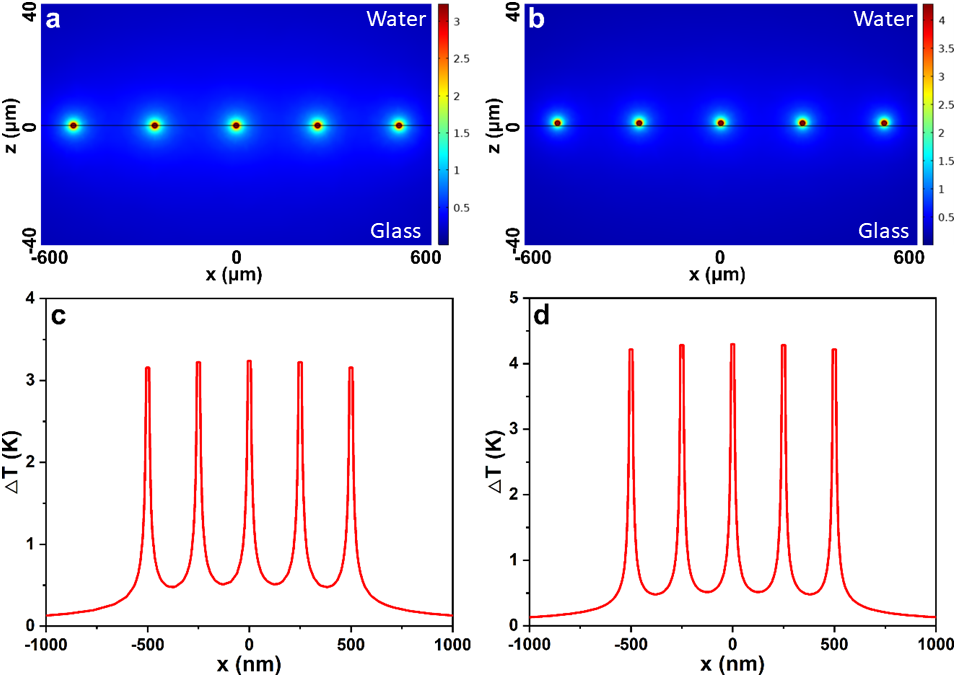
 Figure S4. Heating of 5 gold nanospheres with radius R = 7.5 nm and spatial period p = 250 nm, which are a) half buried in the glass substrate and half immersed in water, and b) loaded on the glass substrate surface and immersed in water. The black lines indicate the glass-water interface. The optical illumination intensity in both cases is 1.27×10^9^ W/m^2^. c & d) Corresponding temperature rise profile along the line across the center of the nanospheres along the x-axis of (a) and (b), respectively.

S2.3 Determination of effective thermal conductivities

In Table S2, we provide the effective thermal conductivities used in our plasmoelectric potential calculations in comparison with Baffou *et al.*’s model.

Table S2. Comparison between calculated effective thermal conductivity with the results obtained following Baffou *et al*.’s proposal.

| Heating configuration | Retrieved $\bar{k}$ (W/(m·K)) | $\bar{k}$ of Baffou *et al*.’s proposal (W/(m·K)) |
| --- | --- | --- |
| Gold dimers on gold film (Section 3.1) | 0.254 | 158.5 |
| Perfect absorbers on silver film (Section 3.2) | 36.86 | 214.5 |
| ITO induced Fano resonance (Section 3.3) | 1.189 | 5.1 |
| Plasmonic lattice mode (Section 3.4) | 7.139 | 5.79 |


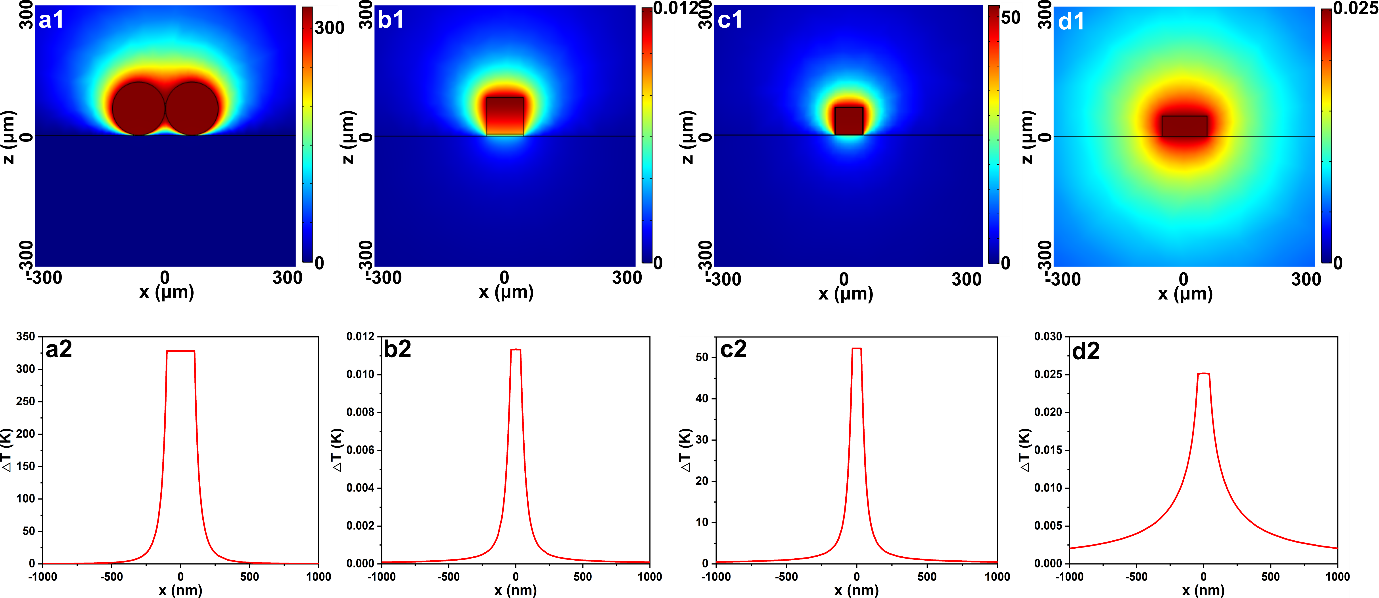


Figure S5. Stationary temperature rise distribution profiles of the four plasmonic nanostructures analyzed in Section 3.1 (a1 & a2, the gold nanosphere dimer coupled to a gold thin film), Section 3.2 (b1 & b2, the perfect absorber consisting of an array of silver nanocubes separated from a silver thin film by an Al_2_O_3_ spacer, Section 3.3 (c1 & c2, the single silver nanocube on ITO), and Section 3.4 (d1 & d2, the array of gold cut wires on an ITO substrate and buried in silica. (a1-d1) are the cut-plane temperature rise distributions in the *y* = 0 plane, while (a2-d2) are the temperature rise distributions along the cut line passing through the nanostructure center in the *x*-axis. The relevant parameters of light illumination and the geometric sizes of plasmonic nanostructures can be found in the main text. The black lines in (a1-d1) indicate the ambient-substrate interface in each structure.

**References**

[S1] Un, I.-W. and Y. Sivan, *The Role of Heat Generation and Fluid Flow in Plasmon-Enhanced Reduction–Oxidation Reactions.* ACS Photonics, 2021. **8**(4): p. 1183-1190.

[S2] Baffou, G., et al., *Photoinduced Heating of Nanoparticle Arrays.* ACS Nano, 2013. **7**(8): p. 6478-6488.
